# Prevalence and burden of rhinitis and rhinoconjunctivitis in 9- to 11-year-old children

**DOI:** 10.1186/2045-7022-3-S2-P6

**Published:** 2013-07-16

**Authors:** Ana Pereira, Ângela Gaspar, Mário Morais-Almeida

**Affiliations:** 1 Hospital CUF-Descobertas, Immunoallergy Department, Lisbon, Portugal

## Background

The aims of this study were: 1) to estimate current rhinitis (CR) and current rhinoconjunctivitis (CRC) prevalence in school aged children using the ISAAC definitions; 2) to characterize children with CR; and 3) to compare children with CR alone with those with CRC symptoms.

## Methods

This was a cross sectional study including a sample of children with 9 to 11 years of age, recruited from 40 randomly selected schools in Lisbon. The ISAAC phase II questionnaire, completed by caregivers, was used to collect data. It included questions on symptoms (wheezing, rhinitis and eczema), use of treatments and health services. Rhinitis ever was defined as the presence, ever, of at least one nasal symptom (sneezing, runny or blocked nose), in the absence of respiratory infection; CR was defined by the presence of rhinitis ever and at least one nasal symptom in the previous 12 months; if CR was accompanied by itchy-watery eyes it was considered that the child had CRC.

## Results

Overall, 1045 children were included; 50% were male, with a mean (SD) age of 9.8 (0.7) years old. The prevalence of rhinitis ever was 36% (n=373) and that of CR was 29% (n=307); 13% (n=135) reported CRC. Children with CR had, on average, 2.7(2.6) months with nose symptoms in the previous year; more than 30% had symptoms in Spring (March (37%), April (33%)) and less than 10% were symptomatic in Summer (July (6%), August (6%)). In the previous 12 month, 57% of the children with CR used medication to treat rhinitis and 9% had at least one visit to a health professional because of nasal symptoms. In children with CR, the previous year's prevalence of eczema was 29% and that of wheezing was 36%. Children with CRC, when compared with those with CR alone, had more rhinitis treatment in the previous year (66% in CRC vs. 51% in CR, p=0.007), had, on average, more months with nasal symptoms (3.4 vs. 2.1 months respectively, p<0.001) and a higher reported impact of nasal disease in daily activities (36% vs. 19%, respectively, reported moderate/severe impact, p<0.001); they also had a significantly higher prevalence of eczema and wheezing (eczema: 41% vs. 21%, respectively, p<0.001; wheezing: 45% vs. 31%, respectively, p=0.009).

## Conclusions

Current rhinitis and rhinoconjunctivitis were highly prevalent in school aged children. The presence of eye symptoms was associated with a greater persistence and impact of nasal disease in daily activities and with a higher prevalence of wheezing and eczema.

